# Supervisor-postgraduate relationship and perceived stress: the mediating role of self-efficacy and the moderating role of psychological resilience

**DOI:** 10.1186/s40359-024-02251-1

**Published:** 2024-12-20

**Authors:** Shen Liu, Xuquan Wang, Han Teng, Wenxiao Gao, Jing Wang, Fan Xu, Minghua Song, Luna Yang

**Affiliations:** 1 Department of Psychology, School of Humanities and Social Sciences, Anhui Agricultural University, Hefei, 230036 China; 2 Institute of Advanced Technology, University of Science and Technology of China, Hefei, China; 3 Mental Health Education Guidance Center, Huzhou University, Huzhou, China; 4 Department of Law, Anhui Vocational College of Police Officers, Hefei, 230036 China

**Keywords:** Supervisor-postgraduate relationship, Perceived stress, Self-efficacy, Psychological resilience, Postgraduate students

## Abstract

**Background:**

Postgraduate studies often entail significant stress, which can profoundly affect students’ well-being and academic performance. The supervisor-postgraduate relationship plays a pivotal role in shaping stress levels among postgraduate students. This study investigates the mediating role of self-efficacy and the moderating influence of psychological resilience in the link between supervisor-postgraduate relationships and perceived stress in postgraduate students.

**Methods:**

This study employed a cross-sectional research design, conducting a survey among 609 postgraduate students selected through random sampling. The participants, aged between 20 and 53 years (*M* = 25.14, *SD* = 3.63), included 265 males and 344 females from various academic stages. Data were collected using validated scales to measure the supervisor-postgraduate relationship, perceived stress, self-efficacy, and psychological resilience. The moderated mediation model analysis was conducted to examine the hypothesized relationships and effects, utilizing SPSS with Hayes’ PROCESS macro to validate the statistical interactions.

**Results:**

The results indicated a significant negative association between the supervisor-postgraduate relationship and perceived stress (*β*=–0.27, *p* < 0.01), with self-efficacy partially mediating this relationship (*β*=–0.14, *p* < 0.01). Additionally, psychological resilience moderated both the direct effect of the supervisor-postgraduate relationship on perceived stress and the indirect effect via self-efficacy (interaction effect *β*=–0.10, *p* < 0.01). These findings underscore the roles of self-efficacy and psychological resilience in reducing stress among postgraduate students, highlighting the importance of supportive supervisory relationships.

**Conclusions:**

These findings underscore the critical importance of supportive supervisor-postgraduate relationships and highlight the roles of self-efficacy and resilience in alleviating stress among postgraduate students. This study offers valuable insights for cultivating positive supervisor-postgraduate relationships and enhancing well-being within postgraduate education.

## Introduction

Postgraduate students face unique stressors that profoundly impact their mental health, including intense academic pressures, financial concerns, and balancing responsibilities across personal and professional domains [1]. Studies consistently show that postgraduate students experience higher rates of anxiety and depression than the general population, with mental health issues affecting nearly 40% of this group [2, 3]. This mental health crisis not only jeopardizes students’ well-being but also threatens their academic and professional development, with potential long-term effects on their career trajectories and overall life satisfaction [1]. The necessity of addressing postgraduate students’ mental health is underscored by the cascading effects that untreated stress and psychological challenges have on academic performance, research quality, and personal relationships. Thus, fostering mental health support for postgraduate students is essential, contributing not only to individual success but also to the broader academic community’s health and resilience. Thus, taking proactive measures to safeguard and enhance the mental health of postgraduate students is imperative. It is essential to promptly implement evidence-based strategies to strengthen postgraduate students’ capacity to manage psychological crises effectively [4]. This approach is vital for cultivating the high-caliber talent required to meet the demands of the modern era.

### Conceptual framework

In graduate education research and management practice, the supervisor-postgraduate relationship is widely recognized as a central focus and key area of discourse [5]. Research indicates that a positive, supportive supervisor-postgraduate relationship is essential for postgraduate students to achieve academic success and maintain strong mental well-being [6]. In recent years, the supervisor-postgraduate relationship has gained increasing attention and emerged as a prominent area of study within graduate education research. However, research suggests that in the day-to-day reality of graduate training, interactions and relationships between postgraduate students and their supervisors are not always smooth or harmonious [7]. In fact, challenges within the supervisor-postgraduate relationship can often become a significant source of stress and strain for postgraduate students. What, then, is the relationship between the supervisor-postgraduate relationship and postgraduate students’ perceived stress? While research has extensively explored postgraduate students’ psychological stress, its sources, and various influencing factors, the specific impact of supervisors on this stress remains an area that warrants further investigation. Notably, there is a lack of in-depth exploration into the underlying mechanisms or pathways through which the supervisor-postgraduate relationship influences postgraduate students’ perceived stress levels.

Perceived stress is the psychological response triggered when an individual encounters potentially threatening stimuli and assesses their ability to manage external pressures or demands effectively [8]. Recently, perceived stress has gained significant attention due to its profound impact on both physical and mental health, correlating strongly with anxiety, depression, and sleep quality—all critical health indicators [9, 10]. When perceived stress surpasses one’s coping capacity, it may impair academic performance and memory function [11]. According to cognitive appraisal theory, both stable personal traits and environmental factors jointly influence one’s perception of stress, potentially leading to adverse outcomes such as depression, anxiety, self-harm, and suicidal ideation [12]. Moderate levels of stress can support adaptability, but excessive, prolonged stress may severely hinder social functioning and overall health. For postgraduate students, the supervisor-student relationship is often their most pivotal interpersonal connection, with recent studies showing it as a potential source of significant psychological distress [13]. Research further demonstrates a strong link between the quality of this relationship and students’ intellectual stress; perceived rejection or exclusion from supervisors can heighten depression and aggression [14, 15]. Thus, fostering a positive supervisor-student relationship can effectively reduce stress and promote psychological stability and healthy development [1]. Building on the above, this study proposes the hypothesis *H*_1_: The quality of the supervisor-postgraduate relationship will negatively predict postgraduate students’ perceived stress levels.

Self-efficacy, an individual’s belief in their own capabilities, plays a key role in managing stress by mobilizing motivation, cognition, and resilience needed to overcome challenges [16]. According to social cognition theory, self-efficacy is shaped by interactions with the environment and others, with strong self-efficacy positively correlated with emotions like hope and pride and negatively correlated with anxiety and frustration [17, 18]. Research indicates that higher self-efficacy bolsters resilience to stress, enabling individuals to persevere in stressful situations and maintain a stable mental state [19, 20]. In graduate education, the supervisor-postgraduate relationship critically influences self-efficacy; supportive mentorship enhances it, while negative interactions may diminish it [21, 22]. A positive supervisor relationship, therefore, not only strengthens self-efficacy but also reduces perceived stress, allowing students to approach academic and personal challenges more confidently. Building on the above, this study proposes the hypothesis *H*_2_: The self-efficacy of postgraduate students mediates the relationship between the quality of the supervisor-postgraduate relationship and their perceived stress.

Psychological resilience refers to an individual’s ability to positive adapt and dynamically adjust when faced with stress or adversity. It embodies the capacity to bounce back and maintain a constructive state following significant challenges [23]. Extensive research has shown that individuals with high levels of psychological resilience tend to demonstrate greater self-awareness and a stronger sense of responsibility [24, 25]. They also possess a wealth of psychological resources, which helps alleviate stress and mitigate its harmful effects [26]. According to the basic psychological needs model, the strength of the impact of a need-supportive environment is moderated by individual psychological traits, such as psychological resilience [27]. Building on the above, this study proposes the hypothesis *H*_3a_: Psychological resilience moderates the relationship between the supervisor-postgraduate relationship and postgraduate students’ perceived stress. A supportive supervisor-postgraduate relationship creates favorable conditions for postgraduate students to pursue independent exploration. However, students inevitably encounter stress throughout this process of self-directed inquiry. Numerous studies have explored the interactive effects of psychological resilience and other protective factors on individuals. For example, psychological resilience can enhance the alleviating impact of perceived social support on loneliness among the elderly in mobile population [28]. Research has shown that psychological resilience amplifies the positive effects of supportive relationships on stress management and well-being. For instance, graduate students with higher psychological resilience demonstrate a more pronounced benefit from constructive supervisor interactions in reducing perceived stress and enhancing academic engagement compared to those with lower resilience levels [29]. Research has shown that interpersonal relationships play a crucial role in college students’ campus life. For instance, strong relationships are also considered a key protective factor for psychological resilience. Building on the above, this study proposes the hypothesis *H*_3b_: Psychological resilience moderates the relationship between the supervisor-postgraduate relationship and postgraduate students’ self-efficacy. The supervisor-postgraduate relationship is a key interpersonal dynamic at the postgraduate stage and is significantly and positively correlated with psychological resilience [30]. Previous research has shown a strong positive correlation between psychological resilience and self-efficacy [31]. Among the various student-teacher relationships, the supervisor-postgraduate relationship is the most direct. Supervisors play a crucial role in the career development of postgraduate students by providing emotional support, intellectual guidance, and social resources [32]. As noted earlier, the supervisor-postgraduate relationship is closely linked to postgraduate students’ self-efficacy. Psychological resilience is a key moderating factor in how the supervisor-postgraduate relationship affects self-efficacy. Psychological resilience theory emphasizes that individuals have the capacity to adapt and transform when facing adversity and stress. The moderating mechanism of psychological resilience can be seen as the result of individuals actively using their internal capacities to adapt and change in response to external stressors [33]. In other words, individuals with high psychological resilience tend to have greater confidence in their ability to undertake certain tasks or complete specific work. They are able to approach self-learning and personal growth with a more positive mindset, actively addressing the various pressures and challenges encountered during their postgraduate journey [34]. Building on the above, this study proposes the hypothesis *H*_3c_: Psychological resilience moderates the relationship between self-efficacy and perceived stress among postgraduate students.

In summary, this study explores the relationship between the supervisor-postgraduate relationship and perceived stress, while also investigating the underlying mechanisms involved. The primary focus of this study is to investigate the mediating role of postgraduate students’ self-efficacy in the relationship between the supervisor-postgraduate relationship and perceived stress. Additionally, it examines how different levels of psychological resilience may moderate both the direct and mediated path in this relationship. This not only enhances our comprehensive understanding of how the supervisor-postgraduate relationship influences perceived stress, but also offers valuable theoretical insights for the development and support of postgraduate students.

## Methods

### Participants

This study adopted a cross-sectional research design and employed a questionnaire-based approach, conducting a randomized survey of postgraduate students. Data collection was carried out through an online platform. Participants were first provided with standardized instructions and an informed consent form, and participation was voluntary. To ensure the randomness of the sample, participants were randomly selected from a pool of postgraduate students through the online platform, ensuring that all individuals had an equal chance of being invited to participate. In total, 687 valid responses were initially collected. Following the data collection, screening questions were employed to identify and exclude 78 invalid responses. This process resulted in a final sample of 609 valid participants, yielding an effective response rate of 88.6%. The final sample included 265 males and 344 females, consisting of 192 first-year postgraduate students, 218 s-year postgraduate students, 149 third-year postgraduate students, and 50 doctoral candidates. The mean age of the participants was 25.14 years, with a standard deviation of 3.63. Regarding inclusion criteria, participants had to be enrolled as postgraduate students at the time of the survey. Exclusion criteria included incomplete responses or failure to meet the specified criteria for postgraduate status. Since all questions in the questionnaire were mandatory fields on the online platform, participants were required to provide complete responses before submitting the survey, resulting in no missing data in the final dataset.

## Measures

### Supervisor-postgraduate relationship scale

This study employed the Supervisor-Postgraduate Relationship Perception Scale, as revised by Yu et al. [35]. The scale comprises 7 items and utilizes a 5-point Likert scale, with 1 indicating “Completely disagree” and 5 indicating “Completely agree”. A higher score on the scale indicates a closer perceived supervisor-postgraduate relationship. In this study, the internal consistency coefficient for the scale was 0.86. The results of the confirmatory factor analysis suggest that the scale possesses strong structural validity, as evidence by the following indices: χ^2^/df = 2.82, RMSEA = 0.055, NFI = 0.977, GFI = 0.984, CFI = 0.985.

### Perceived stress scale

This study utilized the Perceived Stress Scale Short Form, as translated and revised by Chen et al. [36]. The scale comprises 10 items and employs a 5-point Likert scale, with 1 signifying “Completely disagree” and 5 signifying “Completely agree”. A higher score on the scale indicates a greater level of perceived stress experienced by the postgraduate students. In this study, the internal consistency coefficient for the scale was 0.94. The results of the confirmatory factor analysis suggest that the scale possesses strong structural validity, as evidence by the following indices: χ^2^/df = 2.69, RMSEA = 0.053, NFI = 0.980, GFI = 0.973, CFI = 0.987.

### Self-efficacy scale

This study utilized the Chinese Version of the General Self-Efficacy Scale (GSES), as revised by Zhang and Schwarzer [37]. The scale consists of 10 items and employs a 5-point Likert scale, with 1 representing “Completely disagree” and 5 representing “Completely agree”. A higher score on the scale indicates a stronger sense of self-efficacy among postgraduate students. In this study, the internal consistency coefficient for the scale was 0.89. The results of the confirmatory factor analysis suggest that the scale possesses strong structural validity, as evidence by the following indices: χ^2^/df = 2.82, RMSEA = 0.055, NFI = 0.961, GFI = 0.970, CFI = 0.974.

### Psychological resilience scale

This study utilized the Resilience Scale Short Form, as revised by Chen et al. [36]. The scale consists of 10 items and utilizes a 5-point Likert scale, with 1 representing “Completely disagree” and 5 representing “Completely agree”. A higher score on the scale indicates a stronger perceived psychological resilience among postgraduate students. In this study, the internal consistency coefficient for the scale was 0.90. The results of the confirmatory factor analysis suggest that the scale possesses strong structural validity, as evidence by the following indices: χ^2^/df = 2.59, RMSEA = 0.051, NFI = 0.967, GFI = 0.973, CFI = 0.979.

### Data analysis

We conducted a common method bias assessment using Harman’s single factor test. The results indicated that less than 40% of the variance was explained by a single factor, suggesting that there was no significant common method bias present [38]. SPSS 22.0 was utilized to provide demographic variables and descriptive statistics for the four studied variables, as well as to generate correlation matrices. These matrices were used to further control for the variables and to conduct model tests. Model 4 and Model 59 of the PROCESS macro program, developed by Hayes (available for download at http://www.Afhayes.com/), were employed to conduct the moderated mediating model test [39].

## Results

### Common method biases test

All data were collected through participants’ self-reports, which makes the results susceptible to common method bias [38]. Following the approach outlined by Zhou and Long [40], Harman’ s single-factor test was utilized as the method for assessing common method bias. The results indicated that five components had eigenvalues greater than 1.0, with the largest single component accounting for 30.15% of the variance, which is significantly below the 40% threshold. This suggests that the findings of this study did not exhibit any significant common method bias.

### Descriptive analysis

Table 1 presents the correlation matrix of the variables utilized in this study. The results indicated that supervisor-postgraduate relationship was significantly negatively correlated with perceived stress and significantly positively correlated with both self-efficacy and psychological resilience. Perceived stress was significantly negatively correlated with self-efficacy and psychological resilience, while self-efficacy was significantly positively correlated with psychological resilience. These findings suggests that the data obtained in this study are suitable for further analysis.

**Table 1:** Correlation matrices of variables (*n* = 609)

Variables	M	SD	1	2	3	4	5	6
1. Gender	–	–	1					
2. Age	25.14	3.63	–0.07	1				
3. Supervisor-Postgraduate Relationship	3.90	0.72	0.09^*^	–0.10^*^	1			
4. Self-Efficacy	3.82	0.68	0.01	–0.14^**^	0.46^**^	1		
5. Psychological Resilience	3.77	0.72	0.04	–0.05	0.52^**^	0.56^**^	1	
6. Perceived Stress	2.61	0.96	0.12^**^	–0.29^**^	–0.28^**^	–0.38^**^	–0.21^**^	1

Note^*^p-value < 0.05, ^**^p-value < 0.01, and ^***^p-value < 0.001, the same as below

### Moderated mediation modeling testing

To thoroughly investigate the mediating effect of postgraduate students’ self-efficacy between their supervisor-postgraduate relationship and perceived stress, this study utilized Model 4 from the SPSS macro developed by Hayes [39]. During the analysis process, this study included participants’ gender, age, and grade as control variables in the mediation model to ensure the accuracy of the results. The results revealed a significant negative predictive effect of the supervisor-postgraduate relationship on perceived stress (*β*=–0.27, *t*=–7.32, *p* < 0.01). After incorporating the mediating variable of self-efficacy, the supervisor-postgraduate relationship continued to show a significant negative predictive effect on perceived stress (*β*=–0.14, *t*=–3.6, *p* < 0.01). The supervisor-postgraduate relationship exhibited a significant positive predictive effect on self-efficacy (*β* = 0.45, *t* = 12.38, *p* < 0.01), while self-efficacy demonstrated a significant negative predictive effect on perceived stress (*β*=–0.28, *t*=–7.06, *p* < 0.01) (see Table 2). This indicates that the supervisor-postgraduate relationship can negatively predict perceived stress both directly and through the mediating role of self-efficacy. Therefore, postgraduate students’ self-efficacy partially mediates the relationship between their supervisor-postgraduate relationship and perceived stress.

**Table 2:** Mediated modeling testing of self-efficacy

Regression equations		Overall fit index				
Outcome variables	Predictive Variables	*β*	*t*	*R*	*R* ^2^	*F*
Perceived stress	Gender	0.17	4.46^***^	0.42	0.18	43.64^***^
	Age	0.28	7.46^***^			
	Supervisor-postgraduate relationship	–0.27	–7.35^***^			
Self-efficacy	Gender	–0.04	–1.17	0.47	0.22	56.33^***^
	Age	–0.10	–2.83^**^			
	Supervisor-postgraduate relationship	0.45	12.38^***^			
Perceived stress	Gender	0.15	4.29^***^	0.49	0.24	47.60^***^
	Age	0.25	6.90^***^			
	Supervisor-postgraduate relationship	–0.15	–3.67^***^			
	Self-efficacy	–0.28^**^	–7.00^***^			

Subsequently, this study employed Model 59 from the SPSS macro, controlling for participants’ gender, age, and grade as control variables, to explore the moderated mediation model with psychological resilience serving as the moderating variable. The results indicated that after incorporating self-efficacy into the model, the interaction between the supervisor-postgraduate relationship and psychological resilience had a significant positive predictive effect on self-efficacy (*β* = 0.08, *t* = 3.11, *p* < 0.01). The interaction between the supervisor-postgraduate relationship and psychological resilience exhibited a significant negative predictive effect on perceived stress (*β*=–0.10, *t*=–3.20, *p* < 0.01). The interaction between self-efficacy and psychological resilience exhibited a significant negative predictive effect on perceived stress (*β*=–0.11, *t*=–3.80, *p* < 0.01) (see Table 3). This indicates that postgraduate students’ psychological resilience played a significant moderating role in both the direct pathway from their supervisor-postgraduate relationship to perceived stress and the mediated pathway through self-efficacy.

**Table 3:** Moderated mediation modeling testing

	Regression equations		Overall fit index				
Outcome variables	Predictive Variables		*β*	*t*	*R*	*R* ^2^	*F*
Self-efficacy	Gender		–0.02	–0.67	0.61	0.37	70.83^***^
	Age		–0.10	–3.01^*^			
	Supervisor-postgraduate relationship		0.26	6.50^***^			
	Psychological resilience		0.46	12.01^**^			
	Supervisor-postgraduate relationship × Psychological resilience		0.08	3.10^**^			
Perceived stress	Gender		0.13	3.66^***^	0.54	0.29	34.50
	Age		0.24	6.81^**^			
	Supervisor-postgraduate relationship		–0.21	–4.62^**^			
	Self-efficacy		–0.33	–7.14^**^			
	Psychological resilience		–0.01	–0.28			
	Supervisor-postgraduate relationship × Psychological resilience		–0.10	–3.31^**^			
	Self-efficacy × Psychological resilience		–0.11	–3.73^***^			

**Fig. 1:**
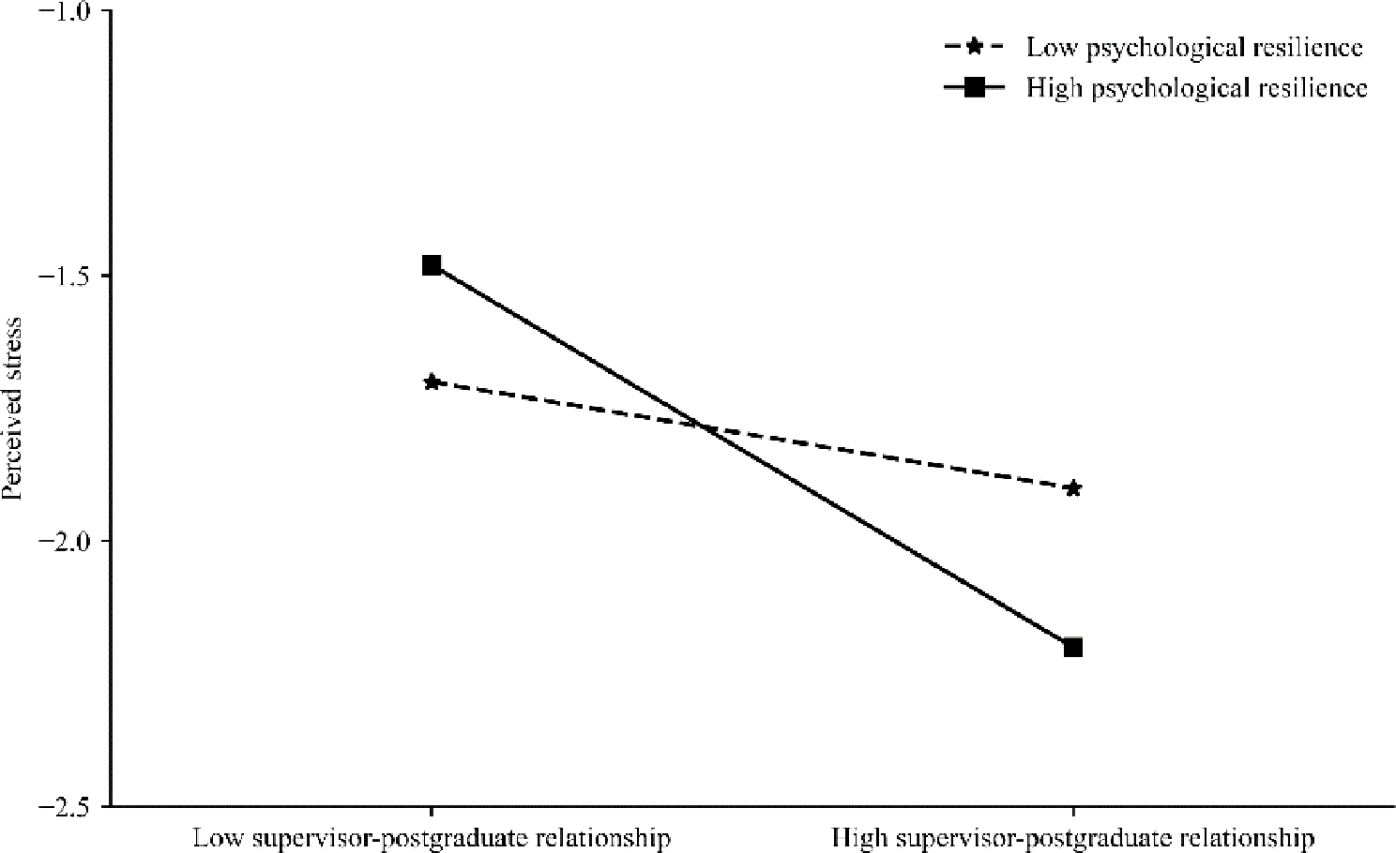
The moderating role of psychological resilience in the relationship between supervisor-postgraduate relationship and perceived stress

To further investigate the moderating effect of psychological resilience at varying levels, this study categorized participants into high and low psychological resilience groups based on the mean value of their psychological resilience, as well as one standard deviation above and below the mean. Using simple slope analysis, this study examined the impact of the supervisor-postgraduate relationship on perceived stress, the influence of the supervisor-postgraduate relationship on self-efficacy, and how self-efficacy subsequently affected perceived stress at varying levels of psychological resilience (see Fig. 1). Based on Fig. 1, for participants with low psychological resilience (below 1 standard deviation of the mean), the supervisor-postgraduate relationship demonstrated a significant negative predictive effect on perceived stress (*B*_simple_=–0.10, *t*=–2.15, *p* < 0.05). For participants with high psychological resilience (above 1 standard deviation of the mean), the supervisor-postgraduate relationship also significantly negatively predicted their perceived stress (*B*_simple_=–0.30, *t*=–4.91, *p* < 0.001), indicating an even stronger predictive effect. This suggests that as the postgraduate students’ psychological resilience increases, the predictive effect of their supervisor-postgraduate relationship on perceived stress also increases, indicating a positive trend.

**Fig. 2:**
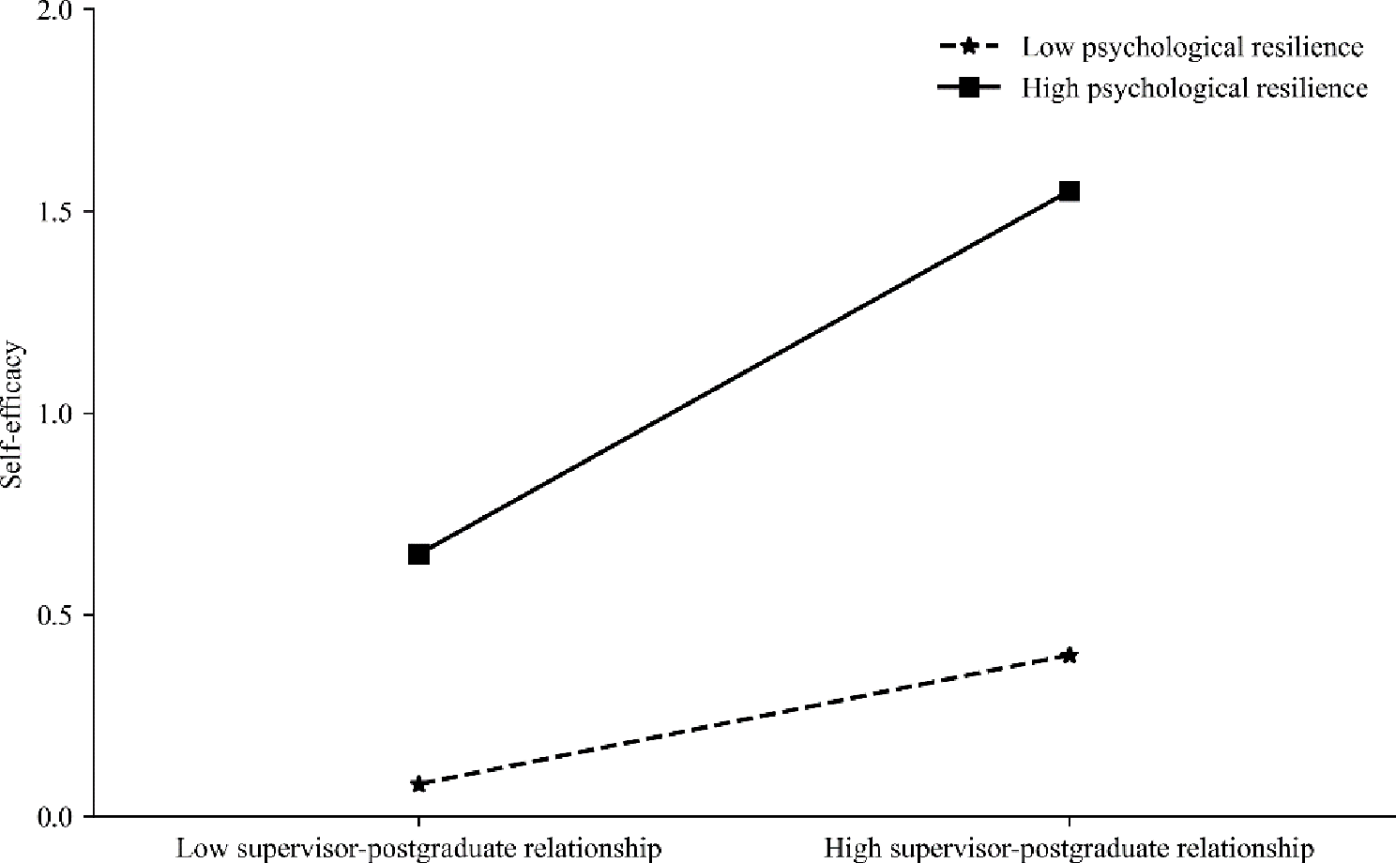
The moderating role of psychological resilience in the relationship between supervisor-postgraduate relationship and self-efficacy

Based on Fig. 2, the influence of psychological resilience on the connection between postgraduate students’ supervisor-postgraduate relationship and their self-efficacy at varying levels is depicted. Specifically, for participants with low psychological resilience (below 1 standard deviation from the mean), the positive predictive effect of the supervisor-postgraduate relationship on their self-efficacy was significant (*B*_simple_=0.17, *t* = 4.13, *p* < 0.001). However, for participants with high psychological resilience (above 1 standard deviation from the mean), the supervisor-postgraduate relationship also significantly negatively predicted their perceived stress (*B*_simple_=0.34, *t* = 6.33, *p* < 0.001), indicating an even stronger predictive effect. This suggests that as the level of psychological resilience increases, the predictive effect of postgraduate students’ supervisor-postgraduate relationship on their self-efficacy also gradually intensifies.

**Fig. 3:**
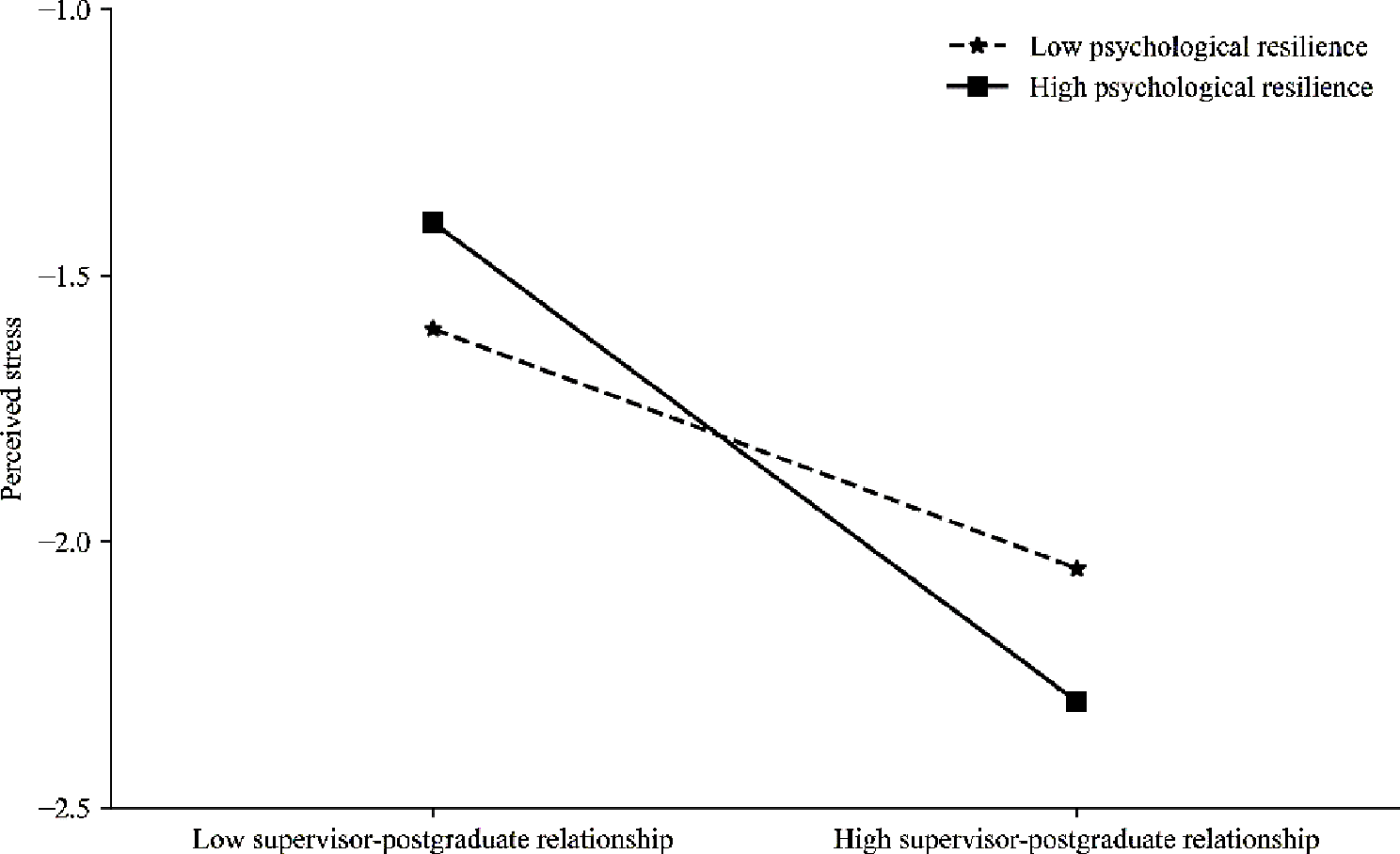
The moderating role of psychological resilience in the relationship between self-efficacy and perceived stress

Based on Fig. 3, compared to participants with low psychological resilience (below 1 standard deviation from the mean), self-efficacy also demonstrated a significant negative predictive effect on their perceived stress (*B*_simple_=–0.21, *t*=–4.57, *p* < 0.001). However, for participants with high psychological resilience (above 1 standard deviation from the mean), this negative predictive effect was more pronounced (*B*_simple_=–0.42, *t*=–7.16, *p* < 0.001). This suggests that as the level of psychological resilience increases, the predictive effect of postgraduate students’ self-efficacy on their perceived stress also gradually increases.

## Discussion

This study, based on social cognition theory, constructed a moderated mediation model in which the supervisor-postgraduate relationship acted as the independent variable, perceived stress served as the dependent variable, self-efficacy functioned as the mediating variable, and psychological resilience was identified as the moderating variable. This study examined the mediating role of self-efficacy in the relationship between the supervisor-postgraduate relationship and postgraduate students’ perceived stress, along with how this mediating effect varied across different levels of individual psychological resilience [40]. The findings hold significance theoretical implications for the study of the supervisor-postgraduate relationship and the psychological health issues faced by students at the postgraduate level, as well as for the development and cultivation of postgraduate students. At the same time, these findings also possess both theoretical and practical significance for advancing national strategies aimed at rejuvenating the country through science and education, building a talent-strong nation, and promoting innovation-driven development [41].

The findings revealed that the supervisor-postgraduate relationship significantly and negatively predicted postgraduate students’ perceived stress. This indicates that when postgraduate students have a positive perception of their relationship with their supervisors, it can reduce their level of perceived stress. Establishing and maintaining a positive supervisor-postgraduate relationship not only reduces postgraduate students’ perceived stress but also fosters their psychological stability and supports healthy development [32]. The relationship between a supervisor and a postgraduate student is a fundamental interpersonal relationship during the postgraduate stage. Maintaining positive interactions and connections with the supervisors may help alleviate communication-related stress among postgraduate students. Additionally, when postgraduate students perceive their supervisors as providing assistance and support in their academic endeavors, it can help alleviate their academic-related stress [42]. Therefore, in guiding postgraduate students, supervisors should prioritize offering both academic and emotional support to cultivate a positive supervisor-postgraduate interactive relationship. At the same time, postgraduate students should also adopt a proactive approach by maintaining open communication and interaction with their supervisors to foster a harmonious supervisor-postgraduate relationship [41]. From a school and social policy perspective, universities and relevant administrative departments should standardize the management of teaching and learning interactions between supervisors and postgraduate students. The goal is to assist postgraduate students in fostering harmonious supervisor-postgraduate relationships, which can alleviate their stress, enhance their psychological well-being, and ultimately improve the quality of higher education.

The mediation analysis results indicated that self-efficacy mediates the relationship between the supervisor-postgraduate relationship and perceived stress among postgraduate students. In other words, when postgraduate students view their relationship with their supervisors positively and constructively, feeling affirmed and supported in their academic endeavors, they cultivate greater confidence in their abilities and a belief in their capacity to successfully complete their studies. This enhancement of self-efficacy subsequently contributes to a further reduction in perceived stress levels. This finding aligns with the predictions of social cognition theory [16] and highlights the underlying mechanism by which the supervisor-postgraduate relationship influences postgraduate students’ perceived stress. During the postgraduate stage, a positive supervisor-postgraduate relationship is a vital factor in enhancing the quality of postgraduate education. Postgraduate students often face a lack of sufficient information or confidence in their ability to succeed in their academic pursuits throughout their studies. The encouragement and support provided by the supervisors play a crucial role in enhancing postgraduate students’ sense of self-efficacy [42]. The enhancement of postgraduate students’ self-efficacy fosters greater confidence in their ability to complete their studies. To some extent, it also empowers them to confront and explore external challenges and changes with a positive and optimistic mindset [43]. This enables them to take timely measures to maintain a balanced state between their internal and external environments, thereby alleviating perceived stress among postgraduate students. This indicates that education administrators should prioritize the significance of the supervisor-postgraduate relationship in the development of postgraduate students [44]. A positive supervisor-postgraduate relationship can not only significantly improve the quality of education but also bolster postgraduate students’ self-efficacy [5]. This enhanced self-efficacy benefits postgraduate students by enabling them to adopt a more proactive coping approach, cultivate a more positive outlook for the future, and ultimately reduce their perceived stress levels more effectively [45].

This study found that both the direct effect of the supervisor-postgraduate relationship on perceived stress and the indirect effect through the mediating role of self-efficacy were moderated by psychological resilience. Psychological resilience, as a positive personality trait, enables individuals to effectively cope with various situations they encounter. This allows them to achieve better adaptation and development, effectively addressing and preventing psychological issues [46]. On the one hand, when postgraduate students encounter an unfavorable supervisor-postgraduate relationship environment, their psychological resilience can help steer them toward positive development [47]. This helps postgraduate students cultivate active and flexible self-regulation, allowing them to adapt positively to their current environmental atmosphere. As a result, they can gradually reduce their perceived level of research-related stress. On the other hand, the positive protective effect of psychological resilience enables postgraduate students to maintain academic enthusiasm and professional engagement, even when they have a poor relationship with their supervisors [48]. This, in turn, helps alleviate their perceived level of stress. At the same time, postgraduate students with high levels of psychological resilience are better equipped to cope with and mitigate the negative psychological effects of a poor supervisor-postgraduate relationship, thereby reducing their perceived stress [49]. Furthermore, psychological resilience significantly moderates the mediating role of self-efficacy. This suggests that psychological resilience can help alleviate postgraduate students’ perceived stress by regulating the mediating pathway of self-efficacy. Research has shown that the moderating mechanism of psychological resilience is demonstrated through its simultaneous protective and compensatory functions [50]. In other words, psychological resilience not only effectively prevents stress caused by external environmental factors such as the supervisor-postgraduate relationship but also decreases postgraduate students’ perceived stress levels by boosting their self-efficacy [34]. Specifically, compared to postgraduate students with low levels of psychological resilience, those with high levels are able to significantly enhance their self-efficacy through the supervisor-postgraduate relationship. This also suggests that individual differences exist in the extent to which a supportive supervisor-postgraduate relationship influences the enhancement of self-efficacy [51]. At the same time, individuals with high psychological resilience are better able to maintain their self-confidence, even when confronted with adverse interpersonal relationships [52]. Furthermore, postgraduate students with higher levels of psychological resilience experience a significant reduction in their perceived stress levels as their self-efficacy improves, in contrast to participants with lower levels of psychological resilience. In other words, psychological resilience has a significant negative moderating effect on the mediating role of self-efficacy in the relationship between the supervisor-postgraduate relationship and perceived stress. This indicates that as an individual’s level of psychological resilience increases, the mediating role of self-efficacy in alleviating perceived stress becomes increasingly pronounced [53]. Therefore, enhancing postgraduate students’ psychological resilience is essential, as it can help prevent a significant rise in their perceived stress levels, even when their relationship with their supervisor is less than ideal.

This study provides a theoretical exploration of the influence of the supervisor-postgraduate relationship on psychological issues among postgraduate students. The findings underscore the significant role of the supervisor-postgraduate relationship plays in influencing students’ psychological issues. The proposed moderated mediation model in this study offers a valuable theoretical framework for understanding the relationship between supervisors and their students. The model enhances the understanding of the nature of the supervisor-postgraduate relationship and its impact on the psychological well-being of postgraduate students. The findings also provide important theoretical implications for how supervisors can effectively guide and support their students. Specifically, the research emphasizes the importance of: (1) Strengthening communication and fostering a healthy supervisor-postgraduate relationship, and (2) Accurately understanding and managing the dynamics of supervisor-postgraduate relationship. By developing a moderated mediation model, this study highlights the importance of enhancing communication and interaction between postgraduate students and their supervisors during the postgraduate stage. Furthermore, the model developed in this study underscores the significance of communication and interaction between postgraduate students and their supervisors, providing compelling empirical evidence for universities and relevant departments.

While this study has yielded meaningful results, it is important to acknowledge the potential presence of several limitations. Firstly, since this study utilized a cross-sectional research design, it may not accurately capture the causal relationship between the supervisor-postgraduate relationship and post students’ perceived stress. To better elucidate this relationship, further longitudinal studies are necessary to validate the findings. Secondly, the survey scale of this study is relatively limited and does not comprehensively encompass postgraduate students from diverse regions and tiers of universities. This limitation, to some extent, affects the generalizability of the research findings. To improve the representativeness and universality of the research, future studies should aim to broaden the scope of the sample. Lastly, the data in this study were primarily derived from self-reported measures by participants, which may introduce subjective biases and impact the objectivity of the assessments related to the relevant variables. To address this shortcoming, future research could consider integrating self-evaluations with other-evaluations (e.g., supervisor or peer assessments) as part of a mixed-methods approach to enhance the accuracy and objectivity of the data. In addition, perceived stress may differ between master’s and doctoral students; however, this study did not distinguish or control for such differences. Future research should take this into account.

## Conclusions

The moderated mediation model developed in this study not only clarifies the mediating role of postgraduate students’ self-efficacy in the relationship between supervisor-postgraduate interactions and perceived stress but also highlights individual differences within this mediating mechanism. This study not only investigates how the supervisor-postgraduate relationship investigates the perceived stress levels of postgraduate students but also examines the variation in the mediating role of this relationship on perceived stress across different levels of psychological resilience. After controlling for participants’ gender, age, and grade, the supervisor-postgraduate relationship was found to significantly predict lower levels of perceived stress among postgraduate students. Self-efficacy partially mediates the relationship between the supervisor-postgraduate relationship and perceived stress. Psychological resilience significantly moderates both the direct path from the supervisor-postgraduate relationship to perceived stress and the indirect path mediated by self-efficacy.

## Data Availability

Data and materials are available on request from the corresponding author.
